# A Renally Excretable Gold Nanoparticle Oncology Platform Enabling Effective Photothermal Therapy and Chemotherapy Combination

**DOI:** 10.1021/acsnanomed.5c00114

**Published:** 2026-01-07

**Authors:** Guojun Xiong, Alexandra Vaideanu, Ryan D. Mellor, Chengwei Jiang, Benjamin Gardner, Nick Stone, Andreas G. Schätzlein, Ijeoma F. Uchegbu

**Affiliations:** † UCL School of Pharmacy, 371646 University College London, London WC1N 1AX, United Kingdom; ‡ School of Physics and Astronomy, 3286 University of Exeter, Exeter EX4 4QL, United Kingdom; § Nanomerics Ltd., London, EC2Y 5 AU, United Kingdom; ∥ Wolfson College, University of Cambridge, Cambridge CB3 9BB, United Kingdom

**Keywords:** PLGA-AuNCs, renal clearance, PTT, tumor ablation, paclitaxel combination therapy

## Abstract

Combination therapies have largely replaced monotherapies in oncology. Thermal therapy has been developed as an adjunctive treatment owing to its safety and high compatibility with established theraypy options. However, bulky equipment and non-standardized protocols have limited its clinical use. The emergence of gold nanoparticle (AuNP)-based photothermal therapy (PTT) offers a simpler approach using injectable formulations and portable near-infrared (NIR) laser devices. However, poor excretion of AuNPs raises safety concerns and hinders translation. Here, we designed a gold nanoplatform that retains the photothermal capacity of AuNPs while enabling efficient excretion. Ultrasmall AuNPs (<6 nm) with a hydrophilic coating may be renally cleared, but clustering normally alters biodistribution and results in long-term hepatic deposition, prior to excretion. In this study, we employed *p*-methoxybenzenethiol (MBT) to stabilize ultrasmall AuNPs, clustered them with poly­(lactic-*co*-glycolic acid) (PLGA), and coated said clusters with acetylated human serum albumin (Ac-HSA). The resulting Ac-HSA-PLGA-AuNCs demonstrated reduced hepatic retention along with renal excretion of ultrasmall gold species. Intratumoral injection followed by 10 min NIR irradiation achieved efficient tumor ablation, outperforming conventional hyperthermia that usually requires hours of heating. Furthermore, combining Ac-HSA-PLGA-AuNC-based PTT with high-dose paclitaxel (160 mg/kg) was safe and yielded enhanced antitumor efficacy. This developed gold nanoplatform offers a promising strategy for translatable PTT-chemotherapy combinations.

## Graphical abstract

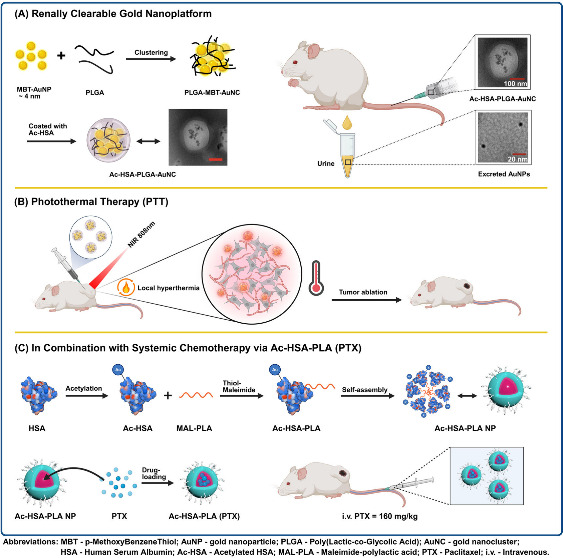

## 1 Introduction

The study of gold nanoparticles (AuNPs) dates back to the 19th century, when Michael Faraday synthesized colloidal gold and observed its characteristic ruby-red color, distinct from bulk gold.^1^ Later studies attributed this phenomenon to localized surface plasmon resonance (LSPR), the collective oscillation of conduction electrons under light excitation.^2^ This unique optical property underpins diverse applications,^3^ notably in photothermal therapy (PTT).^4^ Upon near-infrared (NIR) light absorption, designed AuNPs rapidly convert optical energy into localized heat sufficient to ablate cancer cells while sparing surrounding tissues. For example, the clinical evaluation of gold-silica nanoshells (AuroShell, ∼150 nm) demonstrated the feasibility of exploiting the LSPR properties of AuNPs to achieve controllable thermal ablation of prostate tumors using an NIR laser at 810 ± 10 nm.^5^

However, the chemical inertness of gold results in the poor biodegradability of AuNPs, leading to their long-term accumulation in immune-system organs such as the liver and spleen.^6^ Notably, the nonexcretable nature of AuNPs (>10 nm) can result in prolonged *in vivo* retention, potentially causing chronic inflammation and unforeseen side effects,^7^ which is a key factor limiting the clinical translation of AuNP-based PTT.

To address the excretion issue of AuNPs, researchers have proposed the use of ultrasmall particles (<8 nm), which can be cleared through renal filtration and excreted in urine.^8^ Accumulating evidence demonstrates that ultrasmall AuNPs with a hydrophilic coating (such as glutathione) undergo efficient clearance via glomerular filtration.^9,10^

In 1994, Brust and Schiffrin proposed a typical method of synthesis of uniform ultrasmall AuNPs, named Brust-Schiffrin method.^11^ However, ultrasmall AuNPs prepared with the Brust-Schiffrin method need to be stabilized with alkanethiols (e.g., dodecanethiol) in the oil phase, resulting in hydrophobic AuNPs that would aggregate in aqueous media. Therefore, excretable ultrasmall AuNPs are synthesized using alternative methods^12^ that provide hydrophilic capping, such as by using glutathione (GSH), 4-mercaptobenzoic acid (*p*-MBA), hydrophilic polymers, and proteins.^13^ Promising evidence has shown that nearly 50% of GSH-capped AuNPs (Au25, ∼1 nm) are cleared in mice within 1 day after injection.^14^ Additionally, a water-dispersible ultrasmall AuNP (AuroVist, 1.9 nm) has been commercialized as a CT contrast agent, offering the advantage of renal clearance.^15^

Although ultrasmall AuNPs (<6 nm) with hydrophilic coatings are renally clearable, their LSPR band is typically around 520 nm, which is inadequate for PTT applications, which require absorption above 800 nm.^16^ It has been reported that plasmonic coupling within clustered AuNPs leads to a significant red-shift of the LSPR wavelength,^17^ thereby enabling stronger NIR absorption. In order to apply these excretable ultrasmall AuNPs in PTT, multiple clustering strategies have been developed to enhance their absorption in the NIR region.^10,18,19^ However, the renal clearance efficiencies of these gold nanoclusters (AuNCs), prepared from hydrophilic AuNPs such as GSH-capped,^19^ dextran-coated,^10^ and protein-stabilized ones,^18^ remain low even one month after injection. In fact, the excretion of these AuNCs did not significantly reduce their accumulation in major organs, such as the liver and spleen, even after weeks of injection. This is a significant difference from the reported excretion of GSH-capped ultrasmall AuNPs, of which 50% of the injected dose was cleared within 1 day of injection.^14^ We have noticed that the major biodistribution of these AuNCs is concentrated in the liver and spleen of mice,^10^ which contrasts with the main accumulation of GSH-capped ultrasmall AuNPs in the bloodstream and kidney.^14^ The preferential accumulation of AuNCs in the liver and spleen of mice is also consistent with findings in rhesus macaques, where clustered quantum dots exhibited slow renal filtration and long-term accumulation in the liver and spleen of monkeys.^14,20^ This altered biodistribution of ultrasmall particles, after clustering, may be a key factor contributing to reduced renal filtration, rather than any species differences.

As a result, we hypothesize that clustering ultrasmall AuNPs (coated with hydrophilic ligands) may alter their biodistribution and cause them to accumulate in the liver and spleen, thereby requiring an additional metabolic step in the liver before being excreted in the urine via renal filtration, compared with the original ultrasmall AuNPs. Under this assumption, the ease of declustering and the ability to engage hepatic metabolic pathways to release AuNPs may be important determinants of AuNC clearance. Therefore, in this work, we hypothesize that AuNCs prepared from ultrasmall AuNPs with a hydrophobic but metabolizable coating may be rapidly excreted in the urine following hepatic metabolism. Briefly, *p*-methoxybenzenethiol (MBT) was used in place of alkanethiols in the Brust–Schiffrin synthesis of ultrasmall AuNPs to obtain MBT-coated AuNPs. This design was inspired by the well-characterized metabolic pathway of tramadol,^21^ in which the *p*-methoxyphenyl group undergoes CYP2D6-mediated O-demethylation in the liver to generate a phenolic hydroxyl group, followed by conjugation via uridine diphosphate-glucuronosyltransferase (UGT) enzymes with glucuronic acid or by sulfotransferases with sulfate. These metabolic reactions rapidly produce hydrophilic metabolites of tramadol that are efficiently excreted via the urine. We therefore hypothesize that the *p*-methoxyphenyl moiety of MBT may undergo a similar hepatic metabolic transformation, ultimately leading to increased hydrophilicity and efficient renal clearance of the ultrasmall AuNPs. Subsequently, we used the biodegradable polymer PLGA (poly­(lactic-*co*-glycolic acid)) to cluster MBT-AuNPs into PLGA-MBT-AuNCs through noncovalent interactions. The resulting AuNCs were further coated with a protein, acetylated human serum albumin (Ac-HSA), to obtain a novel AuNC formulation termed Ac-HSA-PLGA-AuNC. In addition to providing a biological coating, Ac-HSA offers a unique advantage by triggering CD44-mediated endocytosis, which significantly enhances the cellular uptake of nanoparticles in CD44 positive (CD44+) cancer cells.^22^ The underlying CD44 uptake mechanisms and supporting data have been extensively described in our previous study.^22^ In this work, we demonstrate that the intravenously injected Ac-HSA-PLGA-AuNCs may be processed by the liver and subsequently excreted in the urine of mice. Meanwhile, PTT was also achieved by intratumoral injection of Ac-HSA-PLGA-AuNCs followed by NIR laser irradiation (808 nm). In addition to achieving successful PTT in mice, a potential synergistic effect was found when combining it with a first-line chemotherapy, and the mice tolerated it well even at a paclitaxel dose of 160 mg/kg. In summary, this work demonstrates a hydrophobic gold nanoplatform that achieves efficient renal clearance, PTT and shows a high compatibility with chemotherapy.

## 2 Results and Discussion

### 2.1 Preparation of Ac-HSA-PLGA-AuNCs

#### 2.1.1 Synthesis of MBT-Capped AuNPs

In this work, we adapted the Brust-Schiffrin method to prepare hydrophobic ultrasmall AuNPs stabilized by *p*-methoxybenzenethiol (MBT) instead of alkanethiols. As illustrated in Figure 1A, the Au (III) chloride was transferred from the aqueous phase (distilled water) to the oil phase (toluene) in the presence of tetraoctylammonium bromide (TOAB). The reducing agent, sodium borohydride (NaBH_4_) water solution, was then slowly added to this two-phase system to achieve homogeneous reduction and obtain uniform ultrasmall AuNPs (stabilized by TOAB). Subsequently, upon the addition of excess MBT, the ultrasmall AuNPs were stabilized in the oil phase via stronger Au–S interactions with MBT, replacing the electrostatic stabilization previously provided by TOAB.^11^ The resulting MBT-capped AuNPs exhibit spherical and ultrasmall dark dots in the transmission electron microscopy (TEM) image, with an average diameter of 3.8 ± 0.8 nm, as shown in Figure 1B and C. Due to the high electron density and strong Z-contrast of gold,^23^ AuNPs appear as dark, high-contrast dots in TEM images.^24^ The hydrodynamic diameter of the MBT-AuNPs was determined to be 4.8 ± 0.6 nm, as shown in the size distribution graph of MBT-AuNPs dispersed in chloroform at a concentration of 0.1 mg/mL (Figure 1D). The ultraviolet–visible (UV–vis) spectrum (Figure 1E) shows a maximum absorption wavelength of approximately 520 nm and weak absorption in the NIR region (>800 nm), consistent with the spectral characteristics of ultrasmall AuNPs reported in other study.^16^ The limited NIR absorption of ultrasmall AuNPs further supports the need for enhancing their NIR absorption for effective PTT applications.

**Figure 1:**
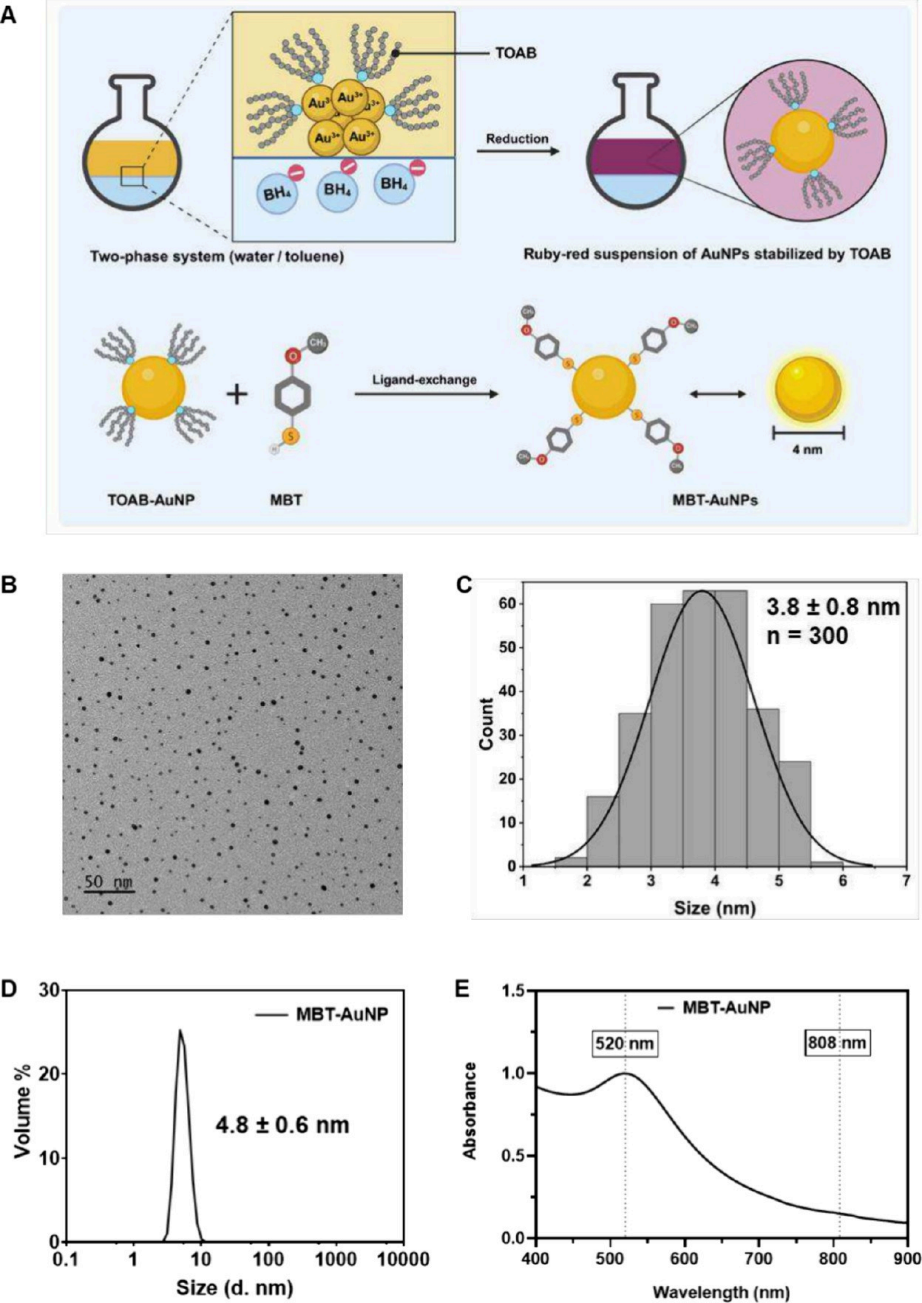
Synthesis and characterization of MBT-AuNPs. (A) Schematic illustration of the preparation of MBT-coated ultrasmall gold nanoparticles (MBT-AuNPs). A two-phase Brust–Schiffrin method (water/toluene) was used to synthesize TOAB-stabilized AuNPs, followed by ligand exchange with 4-methoxybenzenethiol (MBT) to obtain ∼4 nm MBT-AuNPs. Created with biorender.com. (B) TEM image of MBT-AuNPs, showing uniform dispersion (scale bar: 50 nm). (C) Particle size distribution histogram derived from TEM analysis (*n* = 300), indicating an average size of 3.8 ± 0.8 nm. (D) Hydrodynamic diameter distribution of MBT-AuNPs in chloroform. (E) UV–vis spectrum of MBT-AuNPs in chloroform, showing a maximum absorption at 520 nm and limited absorption in the NIR region.

#### 2.1.2 Clustering MBT-AuNPs with PLGA

PLGA is a widely used biocompatible and biodegradable polymer approved by the United States Food and Drug Administration (U.S. FDA) for various biomedical applications.^25^ It is composed of hydrophilic monomers (glycolic acid) and hydrophobic monomers (lactic acid). This amphiphilic nature also makes PLGA suitable for delivering hydrophobic drugs.

In this work, we developed a new clustering approach using PLGA to cluster hydrophobic ultrasmall AuNPs via noncovalent interactions. As briefly illustrated in Figure 2A, a chloroform suspension of MBT-AuNPs was mixed with a PLGA solution in chloroform, and the mixture was subsequently evaporated under a fume hood or with a rotary evaporator to form a thin film containing both PLGA and MBT-AuNPs. The resulting thin film was then dispersed in a polar organic solvent, such as dimethyl sulfoxide (DMSO), which facilitated the clustering of MBT-AuNPs with the hydrophobic segments of PLGA. After probe sonication, the PLGA-MBT-AuNCs were homogeneously dispersed in DMSO. The enhanced NIR absorption (808 nm) and the color change from the ruby red of MBT-AuNPs to the black of PLGA-MBT-AuNCs further confirm the successful clustering of MBT-AuNPs, as shown in Figure 2B. By adjusting the ratio between PLGA and MBT-AuNPs, the NIR absorption (808 nm) of PLGA-MBT-AuNCs may be enhanced accordingly by adding more MBT-AuNPs. As shown in the UV–vis spectrum of PLGA-MBT-AuNCs (Figure 2B), the formulation with 60% PLGA and 40% MBT-AuNPs exhibits a significant enhancement in NIR absorption (808 nm) compared to those with 80% PLGA/20% MBT-AuNPs and 90% PLGA/10% MBT-AuNPs. A higher NIR absorption (808 nm) of AuNCs indicates the capacity for more efficient photothermal conversion.^26^ Meanwhile, this enhancement in NIR absorption (808 nm) was also reproduced by clustering MBT-AuNPs with PLGA of other molecular weights (30 kDa and 50 kDa), showing a similar trend in which a higher proportion of MBT-AuNPs led to greater NIR absorption in the resulting PLGA-MBT-AuNCs. Additional PLGA:MBT-AuNP ratios of 10:0.5, 5:0.5, 5:1, and 6:2 were also evaluated, and their UV–vis spectra are provided in Figure S1. We observed that the enhancement in NIR absorption (808 nm) for the 6:4 formulation was comparable to that of the 6:2 formulation. Therefore, the 60:40 ratio of PLGA to MBT-AuNPs was chosen for the next step of hydrophilic surface coating. Future work will investigate NIR absorption enhancement at higher ratios of MBT-AuNPs and reproduce this effect in AuNPs capped with other ligands in the presence of PLGA.

**Figure 2:**
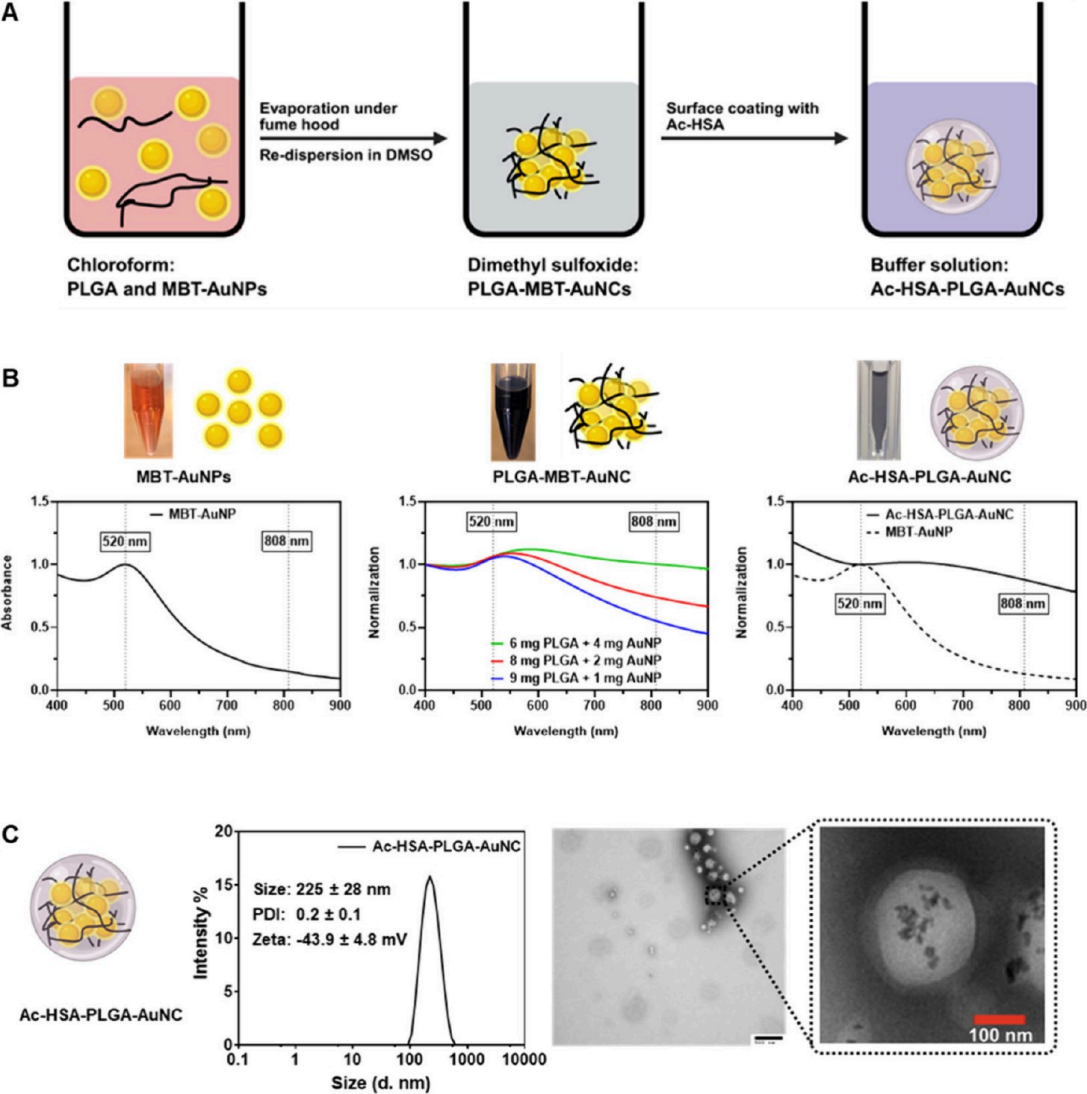
Preparation and characterization of Ac-HSA-PLGA-AuNCs. (A) Schematic illustration of the preparation process: MBT-coated ultrasmall gold nanoparticles (MBT-AuNPs) and PLGA polymer were first codissolved in chloroform. After solvent evaporation and redispersion in DMSO, spontaneous clustering occurred to form PLGA-MBT-AuNCs. These clusters were then coated with acetylated human serum albumin (Ac-HSA) in aqueous buffer to form a stable and biocompatible formulation, Ac-HSA-PLGA-AuNCs. (B) UV–vis spectra and visual appearance of MBT-AuNPs, PLGA-AuNCs, and Ac-HSA-PLGA-AuNCs. The formulation color evolved from ruby red (MBT-AuNPs in chloroform) to black (PLGA-AuNCs in DMSO) and then to translucent gray (Ac-HSA-PLGA-AuNCs in water), corresponding to changes in their optical properties. Notably, clustering of MBT-AuNPs led to red-shifted and broadened plasmon resonance peaks with significantly enhanced near-infrared (NIR) absorption, due to strong plasmonic coupling between closely packed gold cores. (C) Size distribution and morphology of Ac-HSA-PLGA-AuNCs. Dynamic light scattering (DLS) analysis revealed a narrow hydrodynamic diameter distribution (225 ± 28 nm) with a low polydispersity index (PDI) of 0.2 ± 0.1, indicating good colloidal uniformity. The zeta potential was −43.9 ± 4.8 mV, confirming the negatively charged surface, which is beneficial for biological applications. TEM imaging further confirmed the spherical nanocluster morphology and the presence of embedded ultrasmall gold nanoparticles. Created with biorender.com.

#### 2.1.3 Coating of Ac-HSA onto the Surface of PLGA-MBT-AuNCs

Ac-HSA refers to the acetylated human serum albumin. One significant advance of Ac-HSA is its ability to bind to CD44 receptors and trigger CD44-mediated endocytosis of Ac-HSA-based nanoparticles, compared to plain HSA.^22^ Detailed characteristics have been reported in our previous study.^22^

The Ac-HSA was used in this work as a hydrophilic coating material to provide a net negative surface charge and thereby stabilize the PLGA-MBT-AuNCs in aqueous medium for biological applications. The resulting Ac-HSA-PLGA-AuNCs present as a gray-black nanosuspension in water and show enhanced NIR absorption (808 nm) compared to MBT-AuNPs in their UV–vis spectra (Figure 2B). The hydrodynamic diameters, zeta-potentials and polydispersity index (PDI) values of Ac-HSA-PLGA-AuNCs were determined to be 225 ± 28 nm, −43.9 ± 4.8 mV and 0.2 ± 0.1, respectively. As presented in the TEM image (Figure 2C), the dark dots represent the MBT-AuNPs, which are clustered in the PLGA cores in the section 2.1.2 and then further coated by Ac-HSA. Furthermore, the visible protein halo surrounding the nanoparticles in TEM image provides additional confirmation of the successful coating with the Ac-HSA. Both size distribution and TEM image confirm that Ac-HSA-PLGA-AuNCs are nanoscale particles. By using Inductively Coupled Plasma Mass Spectrometry (ICP-MS), the gold content in the Ac-HSA-PLGA-AuNCs was found to be 12.7 ± 0.2% w/w. The coating of Ac-HSA was also successfully reproduced with PLGA-MBT-AuNCs prepared with 30 kDa and 50 kDa PLGA, and the resulting Ac-HSA-coated AuNCs similarly exhibited enhanced NIR absorption, as shown in Figure S2.

Overall, the preparation of Ac-HSA-PLGA-AuNCs involves three major steps: synthesis of ultrasmall AuNPs capped with MBT, clustering of MBT-AuNPs with PLGA, and coating of Ac-HSA onto the PLGA-MBT-AuNCs. The preparation of hydrophobic ultrasmall AuNPs via the Brust-Schiffrin method is well-established, and the use of alternative thiol-based ligands (e.g., methylbenzenethiols) in place of alkanethiols has also been reported in previous studies.^27^ Clustering MBT-AuNPs with PLGA in DMSO is achieved by noncovalent interactions through simple, synthesis-free steps, allowing easy reproduction and scale-up. The subsequent hydrophilic coating is also easily achieved via noncovalent interactions, making the entire preparation simple and scalable. Additionally, the capping ligand of the AuNPs and the hydrophilic coating material may be replaced with other suitable components, and the loading of AuNPs is also adjustable. This novel preparation strategy for AuNCs enables further formulation optimization while maintaining flexibility for alternative applications.

### 2.2 Excretion of Ac-HSA-PLGA-AuNCs in Mice

The aim of developing Ac-HSA-PLGA-AuNCs is to enhance the clearance of AuNPs after PTT. Although the renal clearance of ultrasmall AuNPs with hydrophilic coatings is promising, their clusters failed to achieve this efficient clearance. The threshold for renal filtration is reported to range between 6 and 8 nm.^28^ Ultrasmall AuNPs (<6 nm) with hydrophilic coatings, such as GSH or peptides, have been extensively investigated and shown to undergo rapid clearance, with more than 50% of the injected dose excreted in urine within 1 day in mouse models.^14,29^ Additionally, a commercial CT contrast agent, AuroVist (AuNPs with a size of 1.9 nm), also showed rapid accumulation in the mouse bladder within 2 h after intravenous injection. This suggests that the mouse is a suitable model to investigate the clearance of injected AuNPs. However, when these renally clearable ultrasmall AuNPs were clustered into AuNCs, the clearance of AuNCs was slow and was incomplete months later. This is evidenced by several studies,^10,18,19^ in which different strategies were developed to cluster ultrasmall AuNPs with various hydrophilic coatings. Examples include covalent cross-linking of GSH-capped ultrasmall AuNPs,^19^ encapsulating dextran-coated ultrasmall AuNPs within poly­(ethylene glycol)-poly­(ε-caprolactone) (PEG–PCL) nanoparticles,^10^ and seeding hydrophilic ultrasmall AuNPs onto protein-based nanoparticles.^18^ The renal clearance efficiency differs greatly between hydrophilic ultrasmall AuNPs and their clusters, with the former cleared within days and the latter persisting for months.

We have noticed that the *in vivo* biodistribution in mice of hydrophilic ultrasmall AuNPs is significantly different to their cluster. Hydrophilic ultrasmall AuNPs injected into mice mainly accumulate in the systemic bloodstream and kidney,^14^ whereas AuNCs preferentially accumulate in the liver and spleen.^10,19^ Their different *in vivo* fates may explain why hydrophilic ultrasmall AuNPs can rapidly undergo efficient renal clearance. The accumulation of AuNCs in the liver and spleen suggests that metabolic processing and degradation in the liver are required prior to excretion, which may explain why the excretion efficiency of AuNCs is significantly lower than that of ultrasmall AuNPs. This implies that the benefits of surface modification of ultrasmall AuNPs in renal clearance cannot simply be copied to enable the clearance of AuNCs. Therefore, we hypothesize that the disassembly of these AuNCs into hydrophilic ultrasmall AuNPs within other tissue compartments may hinder their entry into the bloodstream, thereby delaying renal filtration.

In this work, as illustrated in Figure 3A, the Ac-HSA-PLGA-AuNCs suspension was intravenously injected into mice at a dose of 500 μg Au per mouse. We also found a significant accumulation of Ac-HSA-PLGA-AuNCs in the liver and spleen, with a small fraction in the kidney after 2 h of injection (Figure 3B), which is consistent with other results within the first day of injection.^10^ The difference is that these Ac-HSA-PLGA-AuNCs underwent a significant decrease in the liver 5 days after injection, from 145.2 ± 10.1 μg/g to 82.7 ± 25.2 μg/g, n = 4, *p* < 0.01. More than 40% of Ac-HSA-PLGA-AuNCs were cleared from the liver in 5 days. This is a positive response and a significant advance in AuNC excretion research. This reduced retention of gold in the liver within a week of injection is notable, as it was rarely achieved in previous excretion studies of AuNCs. These earlier studies showed no significant decrease in the liver levels of Au over the course of a month.^10,19^ Meanwhile, as shown in the Figure 3B, the Au level in the spleen also exhibited a downward trend over 5 days, although the decrease was not significant, from 173.3 ± 45.3 μg/g to 145.5 ± 39.0 μg/g (n = 4, *p* = 0.3875). This slow clearance of Ac-HSA-PLGA-AuNCs in the spleen corresponds to other previous reports.^10,19^ This finding further supports our hypothesis that disassembly of AuNCs in the spleen may not effectively release ultrasmall AuNPs into the systemic circulation, thereby resulting in low renal filtration efficiency. This phenomenon is not only found in AuNC research, but is also present in studies of other ultrasmall particle clusters. For example, ultrasmall quantum dots (∼5 nm) have shown a high renal clearance efficiency in mice, with 60% clearance within 4 h after injection.^9^ The clustered quantum dots also exhibited long-term accumulation in the liver and spleen, with limited clearance even in monkeys.^20^

**Figure 3:**
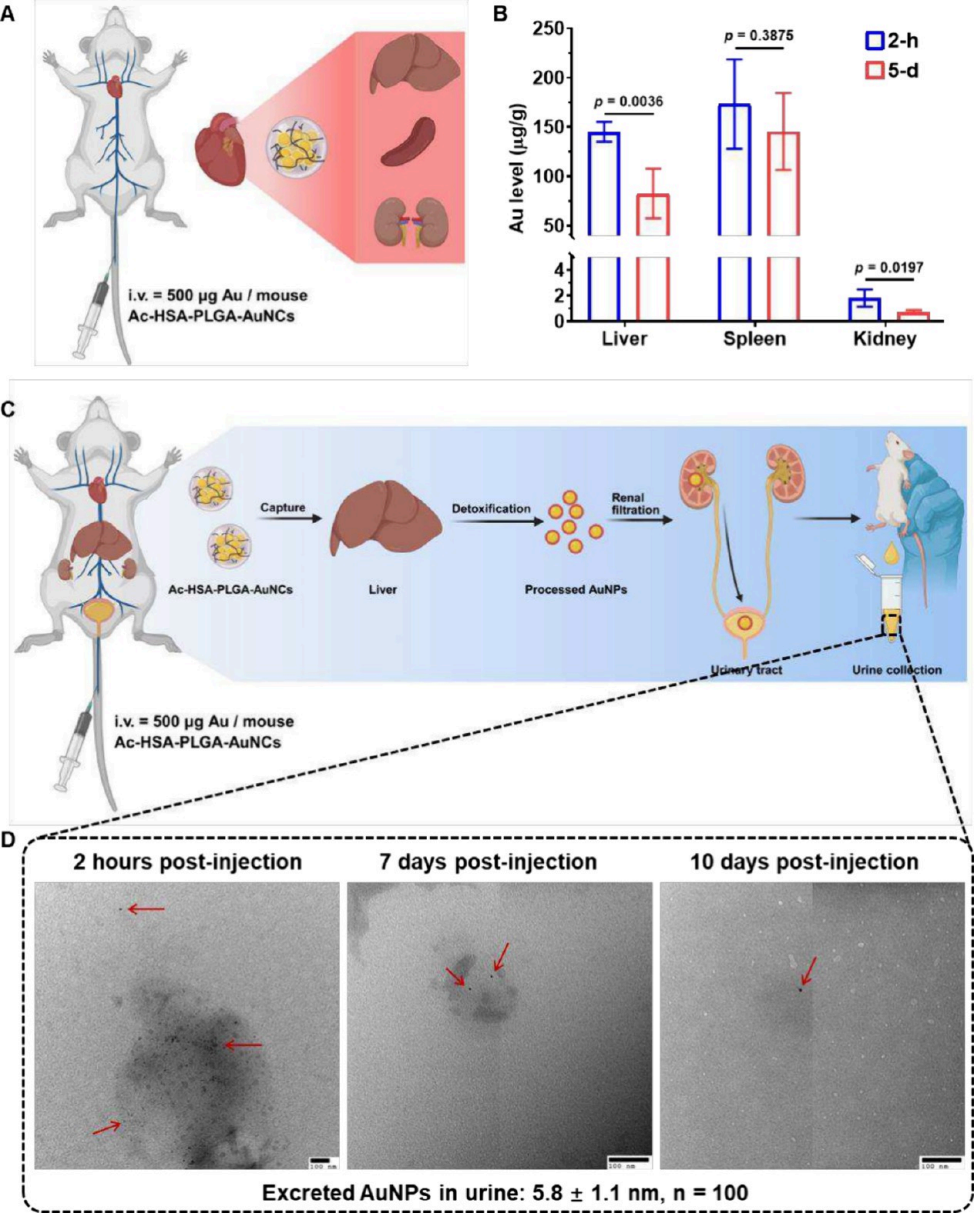
In vivo excretion of Ac-HSA-PLGA-AuNCs. (A) Schematic illustration of systemic nanoparticle distribution following intravenous injection. Ac-HSA-PLGA-AuNCs (500 μg Au per mouse) were administered via tail vein, after which the particles circulated through the heart and distributed systemically, with primary accumulation expected in liver, spleen, and kidney. These organs were subsequently collected for gold quantification. (B) Quantification of gold content in liver, spleen, and kidney at 2 h and 5 days postinjection by ICP-MS. A significant reduction in hepatic gold levels suggests active hepatic processing and partial elimination of nanoclusters over time. Persistent gold signal in the kidney supports its involvement in renal clearance. Data are presented as mean ± SD (*n* = 4). (C) Schematic illustration of the hypothesized clearance mechanism. After hepatic uptake, Ac-HSA-PLGA-AuNCs may undergo metabolic processing and partial disassembly in the liver. The resulting ultrasmall gold nanoparticles may then enter circulation and be cleared via renal filtration into urine. (D) TEM images of urine collected at different time points, showing the presence of ultrasmall gold nanoparticles (indicated by red arrows), confirming renal excretion of metabolically processed AuNPs. Scale bars: 100 nm. Created with biorender.com.

Therefore, we hypothesize that the excretion mechanism of AuNCs may be more complex than that of AuNPs (<6 nm). Simply declustering AuNCs in an acidic environment, such as in lysosomes, may not facilitate the clearance of AuNPs as expected. This is important as current strategies are based on clustering hydrophilic ultrasmall AuNPs, and it is hypothesized that these AuNCs can be declustered in cellular acidic compartments and then cleared via renal filtration.^30^ In fact, previous reports indicate the clearance of these AuNCs was largely hindered and did not occur as intended. Extensive studies have confirmed that the slow clearance of clusters of ultrasmall particles is not due to kidney capacity or species differences.^20,29^ As a result, we hypothesize that the clearance of AuNCs may depend on hepatic metabolism followed by excretion pathways, rather than on simple disassembly of AuNCs in cellular acidic compartments. Based on this rationale, modifying ultrasmall AuNPs with metabolizable ligands may facilitate the rapid excretion of AuNCs rather than simply using hydrophilic ligands. Therefore, we used a hydrophobic and metabolizable ligand (*p*-methoxybenzenethiol) to cap ultrasmall AuNPs. In our hypothesis, as briefly illustrated in Figure 3C, the injected Ac-HSA-PLGA-AuNCs were sequestered by the liver and then underwent disassembly due to the metabolic processes in the liver. The processed AuNPs were finally cleared and excreted via renal filtration. This may explain why there is a significant decline in gold in the liver within 5 days after the injection of Ac-HSA-PLGA-AuNCs. Meanwhile, the slow clearance of Ac-HSA-PLGA-AuNCs in the spleen may be due to the lack of relevant metabolic enzymes and pathways, which contrasts with the enhanced clearance in the liver.

The renally cleared AuNPs in mouse urine were visualized by TEM imaging, as shown in Figure 3D. In this work, we prefer to demonstrate the renally cleared AuNPs with TEM images. It can clearly show that these AuNPs are 5.8 ± 1.1 nm in size, which suggests the injected AuNCs were declustered and renally filtered. This is because several studies have reported the presence of gold originating from injected AuNPs (20–60 nm) in urine and feces.^31,32^ The injected AuNPs (20–100 nm) primarily accumulate in the liver and spleen with little to no reduction for months,^19,31,33^ however, a small fraction can be excreted in feces via the hepatobiliary pathway, and a minor portion of circulating AuNPs may reach the bladder and be eliminated in urine. Therefore, we collected mouse urine and visualized the cleared AuNPs with TEM imaging to provide evidence that their clearance occurred through the renal pathway. We would like to highlight that the number of AuNPs observed in the urine TEM images demonstrates only the presence of excreted particles and does not reflect the renal filtration efficiency, as the urine samples were collected at random time points. The excreted ultrasmall AuNPs were consistently detected in mouse urine for 10 days after injection, with additional supporting evidence provided in the Figure S3. These TEM images may further support our hypothesis that the excretion of AuNCs may be driven by liver metabolism. This is because the clustered ultrasmall AuNPs were hydrophobic in this work, which is different from other studies using hydrophilic ultrasmall AuNPs. Simply declustering Ac-HSA-PLGA-AuNCs into ultrasmall AuNPs can only produce hydrophobic AuNPs. These MBT-coated ultrasmall AuNPs need to undergo metabolism or ligand exchange in the liver to become hydrophilic AuNPs and then be excreted in urine. This design is different from current strategies for developing excretable AuNCs that focus on surface modification of ultrasmall AuNPs for improved renal clearance. We instead proposed that the ease of metabolism of ligands on ultrasmall AuNPs may affect the excretion of their AuNCs. We are not the first group to use hydrophobic AuNPs in the design of AuNCs. Al Zaki et al. clustered dodecanethiol-capped AuNPs (0.9 and 5 nm) using a PEG–PCL copolymer.^34^ In their results, both AuNCs prepared from ultrasmall AuNPs (0.9 and 5 nm) showed a nonsignificant decrease in the liver over a 1-month period following injection.^34^ Meanwhile, limited Au content was detected in the urine (less than 2% of the injected dose) in their work.^34^ Their work demonstrates a significant conclusion that the hydrophobicity of the ligand and the particle size of AuNPs (0.9 nm vs 5 nm) are not the predominant factors in the excretion of AuNCs. The key difference between our work and their study lies in the hydrophobic ligands: we used *p*-methoxybenzenethiol and they used alkanethiols of dodecanethiol. The metabolic pathways of *p*-methoxybenzene are well-established, such as O-demethylation and glucuronidation.^21^ Metabolic reactions often rely on electronegative atoms (such as oxygen and nitrogen) to drive biochemical reactions by creating differences in electron distributions.^35^ Therefore, the metabolism of alkanes, which lack electronegative atoms,^36^ especially for long-chain alkanes (e.g., dodecane), will be more difficult^37^ because the major enzymes (alkane monooxygenases) are expressed in bacteria.^36^ Thereby, we hypothesize that the MBT ligand coated on AuNPs may be metabolizable and able to facilitate the AuNPs passing through the metabolic pathways and finally entering the excretion pathways.

In this work, we have confirmed that the injected Ac-HSA-PLGA-AuNCs can be cleared from mice and excreted via renal filtration in the urine as free ultrasmall AuNPs. Therefore, Ac-HSA-PLGA-AuNCs are worth testing in the application of thermal therapy. In future work, we will systematically investigate how the ligand affects the excretion of AuNCs by using different ligands such as 4-mercaptophenol, 2-mercaptoethanol, and 4-aminothiophenol. Meanwhile, future work will focus on direct identification of hepatic metabolites (e.g., LC-MS/MS), which was beyond the scope of this initial proof-of-concept study.

### 2.4 Efficacy and Safety of Ac-HSA-PLGA-AuNCs in Photothermal Therapy and Combination Chemotherapy

Photothermal therapy (PTT) is a derivative form of thermal therapy, or hyperthermia, in which localized heating is generated by photothermal agents under NIR irradiation.^38^ Thermal therapy has been used as an adjuvant therapy in addition to standard cancer treatments, including chemotherapy and radiotherapy.^39^ By increasing the temperature of targeted tumor tissues and thereby killing cancer cells, a therapeutic effect can be achieved.^40^ Several thermal therapies have been approved for clinical use and have demonstrated synergistic benefits when combined with established treatments.^41^ However, the wide use of hyperthermia in real-life clinical practice remains limited when compared to chemotherapy and radiotherapy. In addition to the high cost of equipment,^42^ the complexity and lack of standardized procedures in hyperthermia pose challenges of interoperator variability and may result in inconsistent or suboptimal therapeutic efficacy when applied differently.^43^ These obstacles may limit the wide application of current hyperthermia in cancer therapy.

Applying AuNP-based PTT may address the major limitations of hyperthermia in cancer therapy. As illustrated in Figure 4A, PTT can be achieved by simply injecting photothermal agents (Ac-HSA-PLGA-AuNCs) intratumorally, followed by irradiation with an 808 nm NIR laser under predetermined settings (4.5 W/cm^2^, 10 min).^44^ Within the method presented in this work, only an NIR laser generator is required, with no need for other bulky and expensive equipment to achieve PTT. Meanwhile, the minimally invasive intratumoral injection is easily reproduced to minimize interoperator variability, and NIR light can penetrate tissues to heat the injected AuNCs effectively. The feasibility and reproducibility of using NIR laser (810 ± 10 nm, ≤6.5 W/cm^2^) to illuminate the selective area of AuNPs, to release heat and achieve a therapeutic effect, have been demonstrated in the clinical evaluation of AuroShells in patients with prostate tumors.^5^ Although not used here, other work in the collaborative team has shown that surface enhanced Raman signals, from labels applied to the gold surface, can provide a direct measure of the temperature local to the gold in real-time.^45^ Used in combination with the work in this paper, such temperature assessments may transform the reproducibility and viability of such treatments.

**Figure 4:**
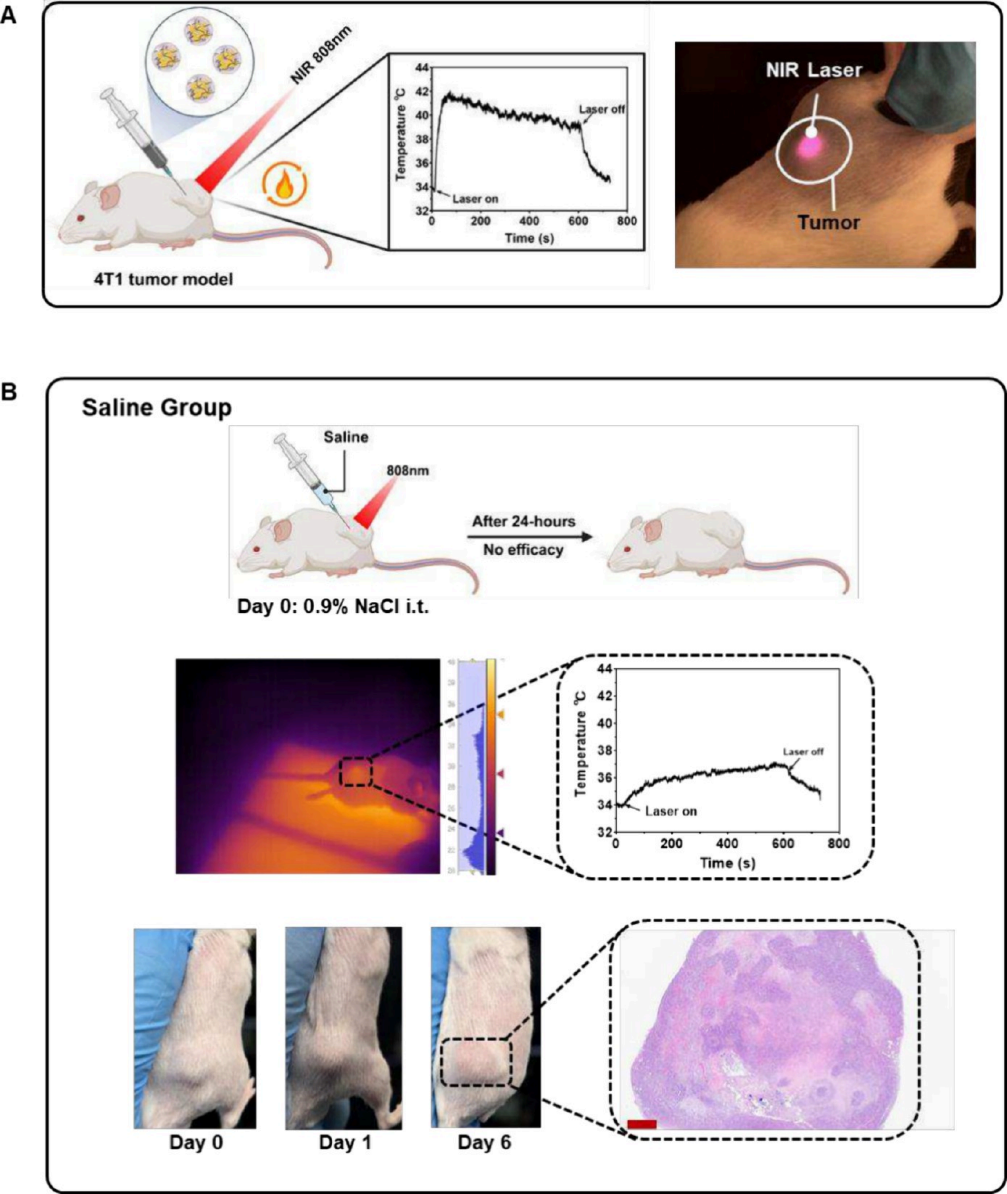
Methodology of photothermal therapy (PTT) and safety of NIR laser irradiation alone. (A) Schematic illustration of the PTT procedure. A photothermal agent was intratumorally injected into 4T1 tumor-bearing mice, followed by near-infrared (NIR) laser irradiation (808 nm, 4.5 W/cm^2^, 10 min) directed at the tumor site. Real-time temperature monitoring was used to confirm whether the tumor region reached the therapeutic temperature (40–42 °C). (B) Overall outcome of the saline control group. Intratumoral injection of saline followed by NIR laser irradiation did not raise tumor temperature to therapeutic levels. Consequently, no photothermal ablation occurred, and H&E staining confirmed intact tumor tissue without necrosis. This demonstrates that NIR laser irradiation alone is safe and has no therapeutic effect. Scale bar: 900 μm. Created with biorender.com.

Irradiation with an NIR laser (808 nm, 4.5 W/cm^2^, 10 min) without NPs present, causes only a limited temperature increase in the illuminated area, which is insufficient to induce protein denaturation or any observable tissue damage. As shown in the saline group (Figure 4B), intratumoral injection of saline followed by NIR laser irradiation (808 nm, 4.5 W/cm^2^, 10 min) did not achieve a therapeutic hyperthermia temperature, reaching only 36 °C. After 24 h of treatment, no signs of skin inflammation, scarring, or thermal burns were observed. Histological examination of 4T1 tumors (Figure 4B) demonstrated intact tumor boundaries, with densely packed cancer cell architecture and large tumor masses, further confirming the absence of tumor ablation in the saline group. These findings confirm that the NIR laser irradiation parameters used in this study are safe and do not cause tissue damage on their own.

In the hyperthermia group (Figure 5), with intratumoral injection of Ac-HSA-PLGA-AuNCs, the temperature in the tumor area illuminated by an NIR laser (808 nm, 4.5 W/cm^2^, 10 min) increased sharply to the therapeutic window of 42 °C for hyperthermia.^39^ One day after this PTT, skin scarring and tumor ablation at the site of NIR irradiation were observed. It has been reported that the intratumoral temperature is higher than the skin-surface temperature detected by infrared camera.^46^ We therefore hypothesize that our infrared camera measured only the skin-surface temperature, whereas the actual intratumoral temperature exceeded the recorded 40–42 °C. The H&E staining of tumor further confirmed the presence of ablated area within the tumor tissue, as highlighted in the orange dashed region, indicating successful photothermal ablation when compared with the saline group (Figure 4B). However, the surrounding, nonirradiated tumor tissue remained viable and continued to grow, highlighting a key limitation of PTT as a monotherapy, without careful control of tumor-wide nanoparticle distribution and light distribution. This localized nature of PTT suggests the added value of combining it with systemic treatments such as chemotherapy, which can suppress residual tumor. The observed tumor skin damage (scarring) represents a reasonable and expected side effect of gold-nanoplatform-mediated hyperthermia, consistent with findings reported in other studies.^16,46^

**Figure 5:**
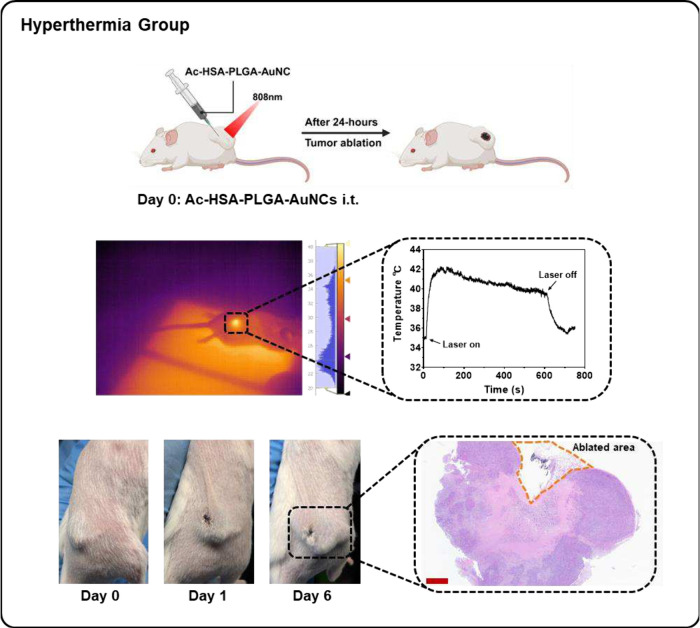
Photothermal ablation of 4T1 tumors induced by Ac-HSA-PLGA-AuNCs under NIR irradiation. Mice bearing 4T1 tumors received intratumoral injection of Ac-HSA-PLGA-AuNCs followed by NIR laser irradiation (808 nm, 4.5 W/cm^2^, 10 min). After 24 h, visible tumor ablation was observed. Real-time infrared thermal monitoring demonstrated a rapid temperature rise at the irradiation site, reaching the therapeutic window of 40–42 °C, thereby confirming that tumor destruction was caused by photothermal heating. H&E staining of tumor sections further visualized the ablated region. Scale bar: 900 μm. Created with biorender.com.

As a result, in addition to AuNP-based PTT, we applied paclitaxel (PTX) chemotherapy as a combination treatment. Taxanes (paclitaxel and docetaxel) are recommended as standard-of-care treatments for both early- and late-stage cancer patients in various national clinical practice guidelines.^47,48^ The PTX used in this study was formulated with the acetylated human serum albumin-polylactic acid (Ac-HSA-PLA) nanoparticles. The preparation of Ac-HSA-PLA (PTX) is briefly illustrated in Figure 6B. In this process, the amine groups of lysine residues on HSA were acetylated via an NHS-ester reaction, yielding acetylated HSA (Ac-HSA). Subsequently, maleimide-polylactic acid (MAL-PLA) was conjugated to Ac-HSA through the released thiol groups, and the resulting Ac-HSA-PLA conjugates self-assembled into nanoparticles. PTX was then encapsulated within these Ac-HSA-PLA nanoparticles. The obtained Ac-HSA-PLA (PTX) nanoparticles were characterized with a hydrodynamic diameter of 177 ± 20 nm, a PDI of 0.06 ± 0.03, and a zeta potential of −34.4 ± 0.9 mV.^22^ As previously reported, Ac-HSA-PLA (PTX) nanoparticles exhibited a superior safety profile in mice, with a maximum tolerated dose (MTD) of 160 mg/kg, compared with the first-generation commercial PTX (Taxol, MTD = 20 mg/kg) and the second-generation commercial PTX (Abraxane, MTD = 120 mg/kg).^22^ Meanwhile, the additional ability of Ac-HSA-PLA nanoparticles to trigger CD44-mediated endocytosis significantly enhances the delivery efficiency toward CD44+ cancer cells as well as cancer stem cells when compared to HSA-based nanoparticles. Therefore, PTX-loaded Ac-HSA-PLA nanoparticles demonstrated significant efficacy in a CD44+ humanized tumor model in which tumors were eliminated and without recurrence after multiple injections when compared to Abraxane, which only showed an inhibition effect.^22^ We thereby hypothesized that multiple injections of Ac-HSA-PLA (PTX) could not only continuously shrink tumor volumes but also eradicate cancer stem cells during this process, achieving complete tumor elimination.^22^ In this study, we applied a single dose of Ac-HSA-PLA (PTX) at its MTD of 160 mg/kg to verify whether Ac-HSA-PLGA-AuNCs driven PTT could be compatible with CD44-targeted PTX chemotherapy, even at such a high dose of PTX. As shown in the hyperthermia plus chemotherapy group (Figure 6), 4T1 tumor-bearing mice received Ac-HSA-PLGA-AuNCs-based PTT on day 0 and localized tumor ablation was observed one-day after hyperthermia (Day 1). Subsequently, these mice were intravenously injected with Ac-HSA-PLA (PTX) nanoparticles at a PTX dose of 160 mg/kg on day 1. Their 4T1 tumor growth was inhibited in the following days. Histological analysis of tumor sections revealed a clear ablation area and reduced tumor size compared to the saline and hyperthermia-only groups. The combination of localized PTT and systemic chemotherapy produced enhanced antitumor efficacy, with PTT ablating the irradiated tumor region and PTX suppressing the growth of residual tumor cells. While an Ac-HSA-PLA (PTX) control was not employed in these studies, as the aim of the studies was to show that excretable gold nanoparticles could produce an antitumor PTT effect (demonstrated by the histology data), it is clear that the combination therapy provides some benefit over the use of gold nanoparticle PTT alone

**Figure 6:**
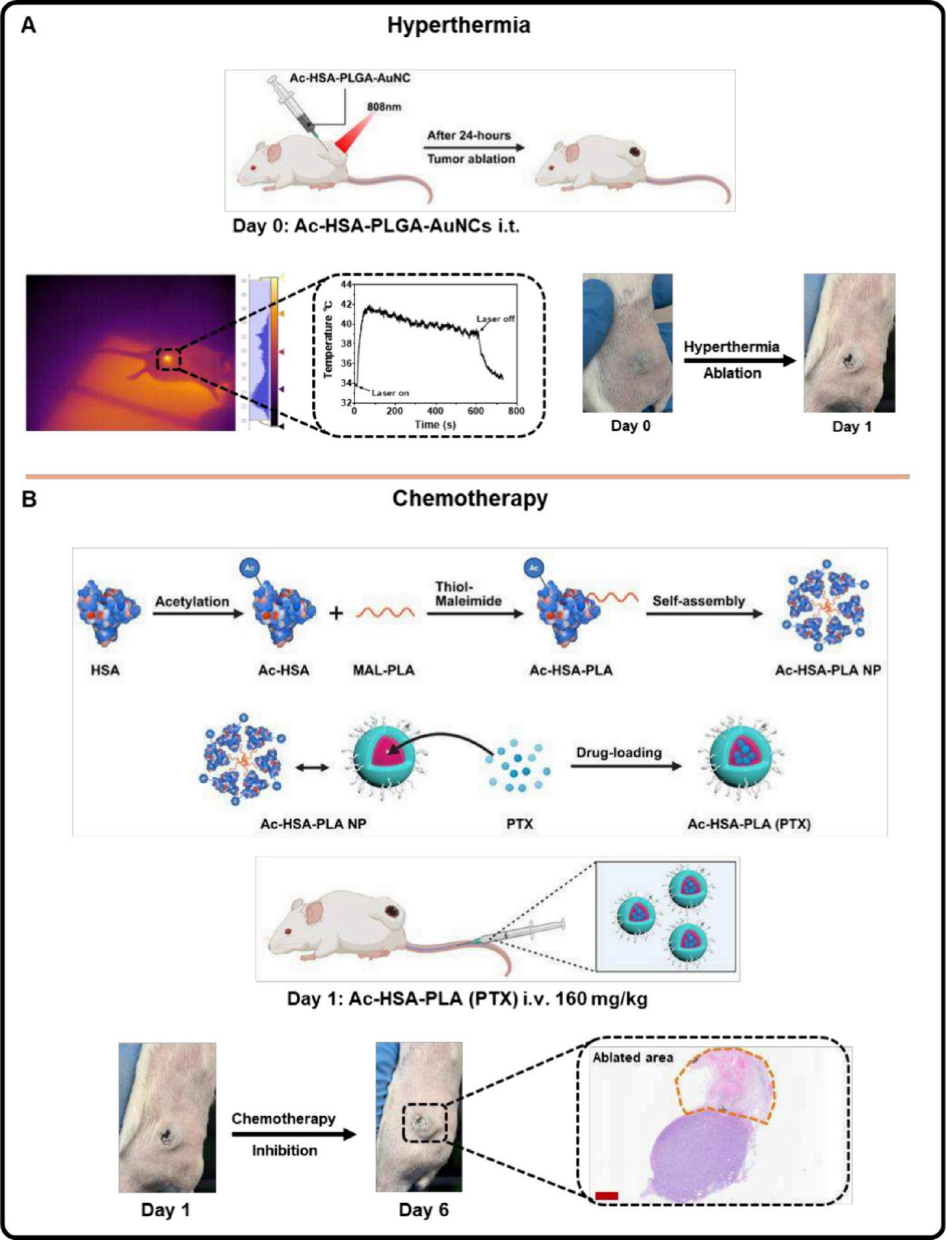
Combination of hyperthermia and paclitaxel chemotherapy using Ac-HSA-PLA (PTX) nanoparticles. (A) Hyperthermia was first applied on day 0 to reproduce the tumor ablation effect of Ac-HSA-PLGA-AuNC-mediated PTT, confirming that elevated tumor temperature (40–42 °C) can induce local tumor destruction. (B) Ac-HSA-PLA (PTX) nanoparticles were developed for systemic paclitaxel (PTX) chemotherapy. Human serum albumin (HSA) was acetylated and conjugated with maleimide-modified PLA (MAL-PLA) via thiol–maleimide coupling, followed by self-assembly into Ac-HSA-PLA nanoparticles. PTX was efficiently encapsulated to obtain Ac-HSA-PLA (PTX) nanoparticles, which were intravenously administered via tail vein at a PTX dose of 160 mg/kg. At the end of the study (Day 6), H&E staining of tumor sections revealed both tumor growth suppression, with smaller tumor size compared to controls, and the presence of thermal ablation regions, demonstrating a synergistic effect of systemic chemotherapy and hyperthermia. Scale bar: 900 μm. Created with biorender.com.

In their tumor volume records (Figure 7A), the 4T1 tumor growth tendency in the untreated group was comparable to that in the saline group, which was intratumorally injected and irradiated with an NIR laser. In the hyperthermia group, driven by intratumoral injection of Ac-HSA-PLGA-AuNCs, tumor ablation was observed; however, the growth of the remaining tumor tissue was unaffected and showed a trend comparable to that of the unheated Ac-HSA-PLGA-AuNCs groups. Both the heated and unheated groups that received intratumoral injections of Ac-HSA-PLGA-AuNCs (Au concentration ≈ 166 μg/mL) exhibited a trend of 4T1 tumor growth inhibition compared with the untreated and saline control groups. This tumor inhibition may be due to the *in vitro* toxicity of AuNCs observed at high concentrations (>100 μg/mL). As shown by the *in vitro* toxicity of Ac-HSA-PLGA-AuNCs (without heating) in both cancerous and noncancerous cell lines (Figure 7C and D), the viability of 4T1 and 293H cells decreased when the Au concentration exceeded 100 μg/mL. This Au-induced cytotoxicity at high concentrations (>100 μg/mL) is also consistent with other reports.^16,49,50^ When PTT was combined with Ac-HSA-PLA (PTX) chemotherapy, tumor ablation was observed and the growth of 4T1 tumors was significantly inhibited compared with that in the other treatment groups (*p* < 0.0001).

**Figure 7:**
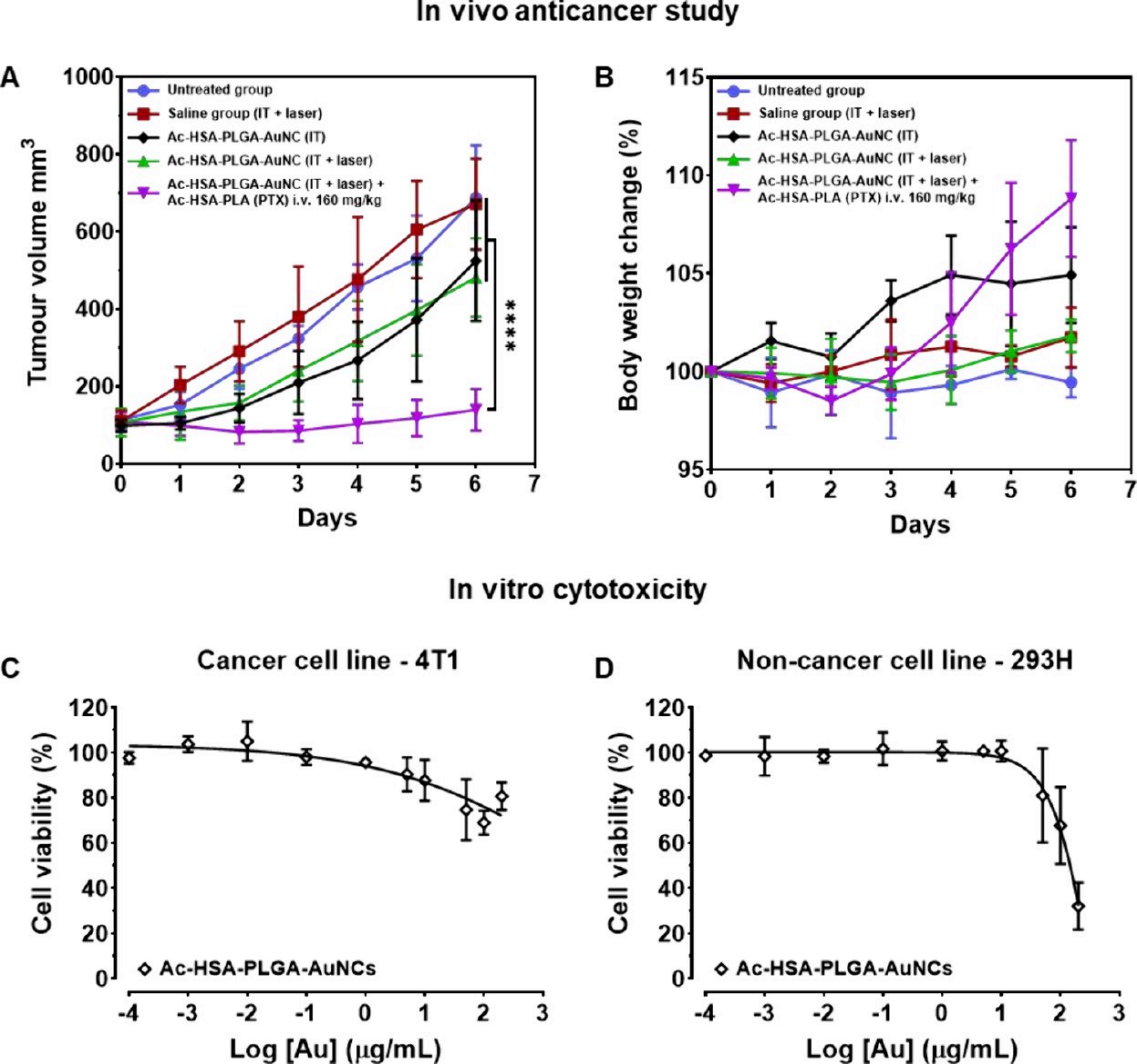
Antitumor efficacy, tolerability, and cytotoxicity of the treatments. (A) Tumor growth curves of the indicated treatment groups (see legend). The combination therapy group exhibited the most pronounced tumor suppression compared with all other groups (*p* < 0.0001). Data are presented as mean ± SD (*n* = 5). (B) Body-weight changes of the same cohorts during the study period, showing no significant weight loss and indicating good systemic tolerability. (C, D) *In vitro* cytotoxicity of the Ac-HSA-PLGA-AuNCs against cancer cells (4T1) and noncancerous cells (293-H). Cytotoxic effects became evident when the Au concentration exceeded 100 μg/mL.

The gross toxicity of these treatments was generally evaluated by monitoring the mouse body weights and by histological examination of major organs at the end of this *in vivo* anticancer study. As shown in their body weight records (Figure 7B), no significant body weight loss was observed from day 0 to day 6. Notably, the body weights of mice were significantly increased after intravenous injection of Ac-HSA-PLA (PTX), rising from 99.6 ± 0.6% on Day 1 to 108.8 ± 3.0% on Day 6 (relative to Day 0, n = 5, *p* < 0.001). This result is consistent with our previous MTD study, in which mouse body weights were significantly increased following intravenous administration of Ac-HSA-PLA (PTX) at a dose of 160 mg/kg.^22^ Histological images (Figure 8) of major organs from mice in the saline group, hyperthermia group, and hyperthermia–chemotherapy combination group revealed no pathological abnormalities in the heart, liver, kidneys, spleen, or lungs. Myocardial fibers, hepatic lobules, renal structures, splenic compartments, and lung alveoli all appeared intact. Both body weight monitoring and histological examination suggest that intratumoral injection of Ac-HSA-PLGA-AuNCs was well tolerated in mice and did not induce severe systemic toxicity. Given the proof-of-concept nature of this study, we limited toxicity evaluation to body weight and histology. Future studies will include comprehensive serum biochemistry analyses, such as alanine aminotransferase (ALT), aspartate aminotransferase (AST), blood urea nitrogen (BUN) and creatinine, to further confirm long-term biosafety.

**Figure 8:**
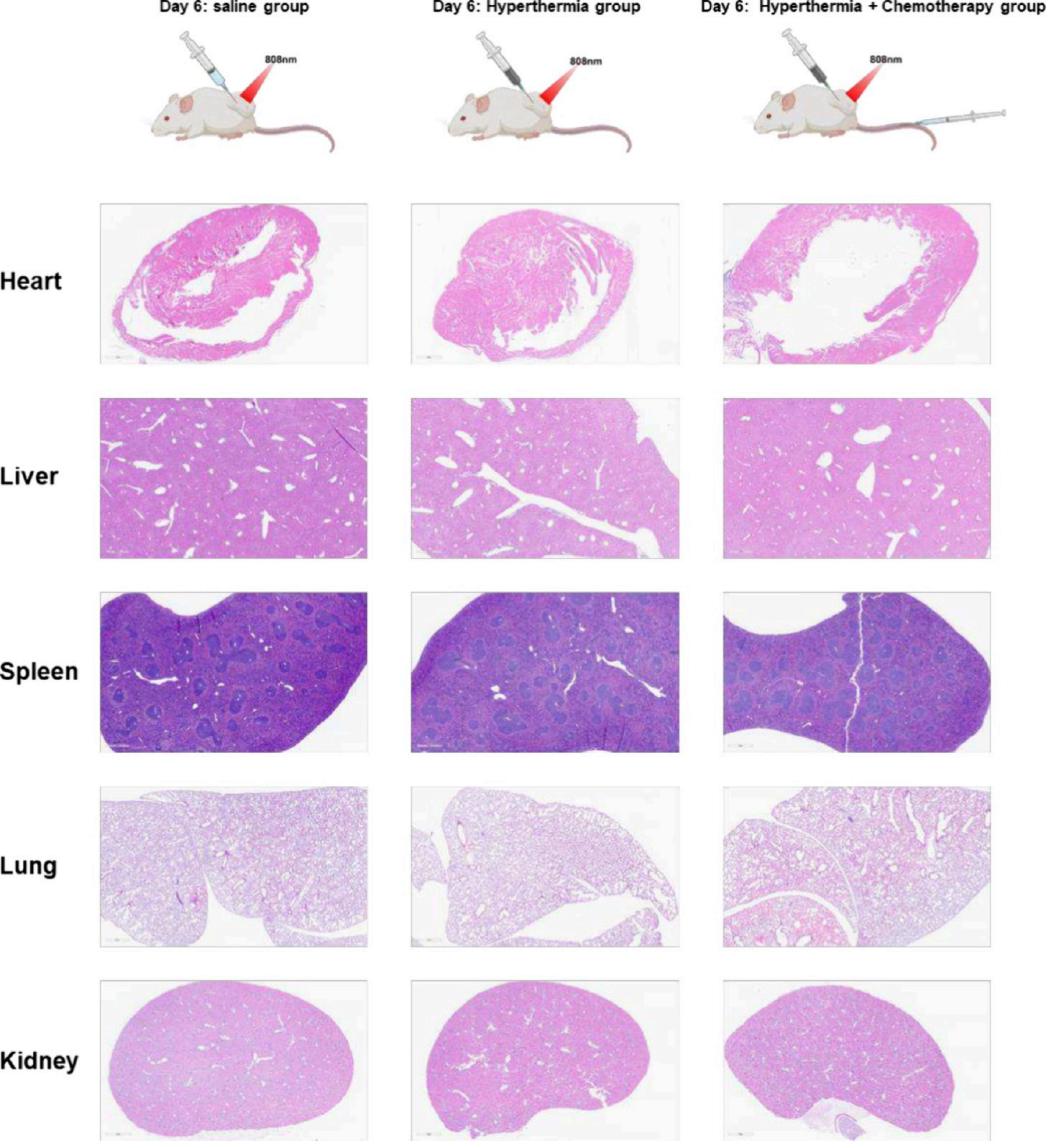
Histological evaluation of major organs following different treatments. Representative H&E-stained sections of heart, liver, kidney, spleen, and lung collected from 4T1 tumor-bearing mice after treatment with saline (control), hyperthermia, or hyperthermia combined with chemotherapy. Compared with the control group, both the hyperthermia group and the combination therapy group showed no evident histopathological abnormalities. These results indicate that, at least at the preliminary stage, systemic toxicity of hyperthermia and chemo–hyperthermia treatment was acceptable, without causing severe organ damage.

As a summary, we achieved an efficient and reproducible PTT in 4T1 tumor-bearing mice with intratumoral injection of Ac-HSA-PLGA-AuNCs and a safe NIR laser (808 nm, 4.5 W/cm^2^, 10 min). Meanwhile, when combining this PTT with Ac-HSA-PLA (PTX)-based chemotherapy, even at a high PTX dose (160 mg/kg), no severe systemic side effects or stressed behaviors of treated mice were observed. This indicates that Ac-HSA-PLGA-AuNC-based PTT represents a promising adjunctive therapy for established treatments.

## 3 Conclusion and Prospectives

In this proof-of-concept study, we developed a novel gold nanoplatform that may provide a new strategy to address the recently proposed challenge of AuNC clearance. Unlike previous studies that relied on hydrophilic ligand coatings, our design appears to exploit hepatic metabolism of MBT to enhance renal clearance, representing a distinct and underexplored strategy. The developed Ac-HSA-PLGA-AuNCs have shown evidence of enhanced hepatic clearance within 5 days of injection, which has been rarely reported in previous research. Our urine TEM images have clearly shown the processed ultrasmall AuNPs, indicating the injected Ac-HSA-PLGA-AuNCs are renally excretable. In the following PTT study, intratumorally injected Ac-HSA-PLGA-AuNCs rapidly and efficiently generated heat under a safe NIR laser to ablate the targeted tumor site. However, only a portion of the tumor was ablated. When combined with PTX chemotherapy, the treatments of PTT and Ac-HSA-PLA (PTX) at a PTX dose of 160 mg/kg were well tolerated by mice. Notably, the MTD of PTX from Abraxane (a second-generation PTX) in mice is 120 mg/kg. This combination therapy exhibited a potential synergistic effect, with PTT ablating localized tumor tissue and systemic PTX suppressing the progression of the remaining tumor. Generally, this novel gold nanoplatform (Ac-HSA-PLGA-AuNC) achieved the major aims of this proof-of-concept study: enhanced renal clearance, effective PTT for tumor ablation, and safety in combination with chemotherapy. These findings may help advance the broader clinical translation of hyperthermia-based therapies and ultimately provide additional therapeutic benefits for cancer patients.

Despite these encouraging outcomes, several aspects of this work warrant further investigation. First, the metabolic fate of MBT-coated AuNPs has not yet been directly characterized by LC-MS/MS or *in vitro* microsomal assays, and future studies will aim to identify specific hepatic metabolites. Second, urine TEM images and ICP-MS quantification provide supportive evidence of renal clearance, but additional pharmacokinetic and cumulative excretion studies would help to more comprehensively establish clearance kinetics. Third, the gross toxicity evaluation in this study was evaluated with body weight monitoring and histological analysis; inclusion of detailed serum biochemistry (ALT, AST, BUN, creatinine) and extended observation will further confirm the long-term biosafety profile. Finally, the therapeutic efficacy was evaluated in murine tumor models, and future studies employing human-derived tumor models will be important to better reflect clinical relevance.

By addressing these points in future projects, this strategy can be further refined and holds promise for eventual clinical translation.

## 4 Materials and Methods

### 4.1 Preparation of Ac-HSA-PLGA-AuNC

#### Step 1: Synthesis of *p*-Methoxybenzenethiol (MBT)-Capped AuNPs

This synthesis method is adapted from the Brust–Schiffrin method.^11^

Chloroauric acid (HAuCl_4_, #223620050, ThermoFisher Scientific, UK, 340 mg) was dissolved in 40 mL of distilled water. Toluene (#T/2250/17, ThermoFisher Scientific, UK, 70 mL) containing tetraoctylammonium bromide (TOAB, #F013939, Fluorochem Limited, UK, 2 g) was added to the chloroauric acid solution and stirred at a speed of 400 rpm in an oil bath at 37 °C. Once all of the chloroauric acid was transferred from the water phase to the oil phase, Sodium borohydride water solution (NaBH_4_, #452882, Sigma-Aldrich, UK, 304 mg, 20 mL) was slowly added to the oil phase and stirred at a speed of 400 rpm in an oil bath at 37 °C. After approximately 5 min of reduction, toluene (20 mL) containing MBT (#109525, Sigma-Aldrich, UK, 2 g) was added and stirred at a speed of 400 rpm in an oil bath at 37 °C for 4 h. The resulting mixtures were transferred to a separatory funnel, water-soluble impurities were removed with washing 5 times with distilled water. The water residue in the toluene phase was dried with excessive magnesium sulfate anhydrous (#793612, Sigma-Aldrich, UK). The MBT-AuNPs in toluene were concentrated by using a rotary evaporator (RV 10 Dry Ice Condenser, IKA, UK) and then precipitated in anhydrous methanol. After centrifugation (10 min, 5000 rpm), MBT-AuNPs were settled in the pellet, and hydrophobic impurities were disposed of in the supernatant. Subsequently, MBT-AuNPs were washed with anhydrous methanol three times and anhydrous ethanol twice to largely remove impurities using this centrifugation method. The resulting MBT-AuNPs were collected and dried in the fume hood overnight. Yield = 116 mg.

#### Step 2: Clustering AuNPs with Poly­(lactic-*co*-glycolic acid), PLGA

The dried MBT-AuNPs were dispersed in a mixture of chloroform and ethanol (CHCl_3_:EtOH = 2:1) at a concentration of 4 mg/mL. PLGA (**#**719897, Sigma-Aldrich, UK, 12 kDa) was dissolved in CHCl_3_ at a concentration of 10 mg/mL. Mixing PLGA (30 mg, 3 mL) and MBT-AuNPs (20 mg, 5 mL), the resulting mixtures were then dried in the fume hood overnight. Dimethyl sulfoxide (DMSO, 2 mL) was added to disperse the clusters of PLGA-AuNPs. To obtain a homogeneous cluster of PLGA-AuNPs, 5 min of probe sonication (5 amplitudes, Soniprep 150plus, MSE, UK) was applied.

#### Step 3: Coating Ac-HSA to PLGA-MBT-AuNCs

The preparation of Ac-HSA was described in the previous work.^22^ The Ac-HSA (50 mg) was dissolved in phosphate buffered solution (0.1 M, pH = 6.8, 10 mL). Subsequently, DMSO (2 mL) containing PLGA-AuNP clusters (50 mg) was added to the Ac-HSA solution. After 2 cycles of probe sonication (9 amplitudes, 5 min), the resulting Ac-HSA-PLGA-AuNC dispersion was dialyzed against distilled water for 24 h (MWCO = 100 kDa) to remove salts, DMSO and free Ac-HSA. The resulting Ac-HSA-PLGA-AuNC nanoparticles were redispersed using bath sonication for 10 min and filtered with a 0.7 μm syringe filter (**#**5190–5122, Captiva Syringe Filters, Agilent, UK). The purified Ac-HSA-PLGA-AuNC was lyophilized and then stored at 4 °C.

### 4.2 Absorption in Ultraviolet–Visible (UV–vis) Regions

The UV–vis spectra of MBT-AuNPs, clusters of PLGA-AuNPs and Ac-HSA-PLGA-AuNCs were obtained using a UV–vis spectrophotometer (UV-1650PC, Shimadzu, Japan) in a wavelength range from 400 to 900 nm.

### 4.3 Transmission Electron Microscopy, TEM

MBT-AuNPs were redispersed in a mixture of CHCl_3_ and EtOH (CHCl_3_:EtOH = 2:1) at a concentration of 0.1 mg/mL. The resulting MBT-AuNP dispersion was dripped onto a TEM grid and left to dry. After imaging, particle sizes (>300 counts) were determined using ImageJ software.

The Ac-HSA-PLGA-AuNC lyophilized powder was redispersed in distilled water at a concentration of 5 mg/mL. The resulting Ac-HSA-PLGA-AuNC nanosuspension was placed onto a TEM grid for 5 min. Subsequently, the TEM grid was dried with tissue paper, and a droplet of neutral phosphotungstic acid solution (#79690, Sigma-Aldrich, UK, 1% w/v) was added for 1 min of staining. After washing with distilled water, the grid was dried with tissue paper.

The prepared TEM samples were stored in a dark location until imaging.

### 4.4 Dynamic Light Scattering, DLS

The hydrodynamic diameters, zeta potentials and polydispersity indices (PDI) of Ac-HSA-PLGA-AuNCs were determined via the DLS technique by using a Malvern Nano-ZS (Malvern Panalytical Ltd., UK). The lyophilized sample (4 mg) was dispersed in distilled water (2 mL). Each sample was measured in triplicate, and the testing was conducted across three different batches of samples.

### 4.5 Quantification of Gold Content in Ac-HSA-PLGA-AuNCs

The Ac-HSA-PLGA-AuNCs were dispersed in distilled water at a concentration of 40 mg/mL. An aliquot (10 μL) of the suspension was digested using an open wet acid digestion approach as follows.

Nitric acid (69% v/v), Hydrochloric acid (HCl, 37% v/v) and hydrogen peroxide (50% v/v) were super purity grade (<1 ppb trace contaminants) and purchased from ROMIL, UK.

Samples were digested with concentrated nitric acid (69%, 4 mL) in borosilicate glass tubes (Fisher Scientific, UK, leached with 1% nitric acid at 60 °C overnight, thoroughly rinsed with Millipore grade water and dried in the air) overnight at room temperature prior to heating up to 80 °C on a heating block overnight. Then, HCl 37% (1 mL) was added, and samples were kept on a hot block for another hour. Digests were allowed to cool to room temperature and then diluted to 50 mL final volume with a 1% l-cysteine (Sigma, UK) 5% v/v HCl solution ensuring that the expected concentration falls within the range of the calibration curve.

The Inductively coupled plasma–mass spectrometry (ICP-MS) used was an Agilent 7900 series (Agilent Technologies, UK), externally calibrated between 0.1 μg/L and 1000 μg/L prepared in 1% m/v l-cysteine 5% HCl v/v from a 1000 mg/L stock in 20% HCl v/v (Alfa Aesar, US). The samples were bracketed by replicates of the lowest and highest concentrations in the calibration curve (0.1 ug/L was recovered at 0.092 ± 0.003 ug/L difference to the expected value −0.16% and 1000 ug/L was measured as 901.6 ± 1.9 ug/L with a difference of 0.5% to the expected value), and 10 μg/L drift and 10 μg/L calibration check solutions were measured every 5 samples for quality control. Between samples, the system was washed by alternating between solutions containing both l-cysteine (1% w/v) and HCl (5% v/v) or HCl (5% v/v) alone, to reduce the Au memory effect which can otherwise lead to anomalously high signal from low concentration samples. ^181^Ta was run to check for interference from ^181^Ta^16^O+. An internal standard (^193^Ir, 250 μg/L) was mixed online with the samples. The ICP-MS instrument settings were as follows: sample uptake rate, 0.3 rps; RF power, 1550 W; Helium collision cell gas flow, 5 L/min; Argon nebulizer gas flow, 1.2 L/min; spray chamber temperature, 2 °C; and integration time, 0.3 s for Au and 0.1 s for Ir. The absolute mass of gold given for each nanoparticle sample was an average of four technical replicates digested from the same homogeneous solution used for injection in vivo.

### 4.6 Biodistribution of Ac-HSA-PLGA-AuNCs in Mice

This animal experiment was conducted in accordance with the UK Animals (Scientific Procedures) Act 1986 and approved by the University College London Animal Welfare and Ethical Review Body (AWERB). This work was carried out under the UK Home Office Project License (PP4970379) and Personal License (I1827609).

Female BALB/c mice (8–10 weeks, 20 ± 2 g, n = 8, Charles River Laboratories, UK) received intravenous injection of Ac-HSA-PLGA-AuNCs at a pure gold dose of 500 μg per mouse. Mice were sacrificed after 2 h of injection (n = 4) and 5 days of injection (n = 4). Their livers, spleens and kidneys were extracted and rinsed with phosphate buffered saline (PBS, pH = 7.4). The organs were weighed and digested with concentrated nitric acid (69%, 4 mL) and hydrogen peroxide 50% (500 μL) in borosilicate glass tubes (Fisher Scientific, UK, leached with 1% nitric acid at 60 °C overnight, thoroughly rinsed with Millipore grade water and dried in the air) overnight at room temperature prior to heating up to 80 °C on a heating block for overnight. Then, HCl 37% (1 mL) was added, and samples were kept on a hot block for another hour. Digests were allowed to cool to room temperature and then diluted to 50 mL final volume with a 1% l-cysteine (Sigma, UK) 5% v/v HCl solution. Liver samples were further diluted 1 in 10 to ensure total mass solids less than 2%. Samples were filtered through 0.22 μm Teflon membranes. Calibration curves, repeats of lowest and highest concentration for accuracy and reproducibility (0.1 ug/L was recovered at 0.095 ± 0.0009 ug/L a difference of −4.2% to expected and 1000 ug/L was measured as 949.3 ± 80.4 a difference of 4.3% to the expected value) and calibration check solutions were ran as described for nanoparticle solutions and ICP-MS (Agilent 7900, Agilent Technologies, UK) settings were also as above. To provide further external validation, oyster tissue NIST1566b (LGC, UK) with a reference value provided by NIST of 0.0106 ± 0.0028 mg/kg (not certified, uncertainty 26.4%) was digested in a similar manner to samples (mass dried tissue used approximately 0.2 g); recovery was 91.5% ± 3.9% (n = 4).

### 4.7 Urine TEM

This animal experiment was conducted in accordance with the UK Animals (Scientific Procedures) Act 1986 and approved by the University College London Animal Welfare and Ethical Review Body (AWERB). This work was carried out under the UK Home Office Project License (PP4970379) and Personal License (I1827609).

Female BALB/c mice (8–10 weeks, 20 ± 2 g, n = 3, Charles River Laboratories, UK) received intravenous injection of Ac-HSA-PLGA-AuNCs at a pure gold dose of 500 μg per mouse. Mouse urine was collected at 2 h, 1 d, 3 d, 5 d, 7 d, and 10 d after injection. The collected urine samples were respectively dropped onto TEM grids and allowed 5 min for attachment. After washing with distilled water, the grids were blotted dry with tissue paper. No staining step was required in this procedure. The prepared TEM samples were stored in a dark location until imaging.

### 4.8 *In Vivo* Antitumor Study

This animal experiment was conducted in accordance with the UK Animals (Scientific Procedures) Act 1986 and approved by the University College London Animal Welfare and Ethical Review Body (AWERB). This work was carried out under the UK Home Office Project License (PP4970379) and Personal License (I1827609).

4T1 cells (**#**CRL-2539, ATCC, UK) were each cultured in a tissue culture flask with a vented cap using a complete medium composed of Advanced RPMI 1640 Medium (**#**12633012, Thermo Fisher Scientific, UK) supplemented with heat-inactivated Fetal Bovine Serum (FBS, #F9665, Sigma-Aldrich, UK, 10% v/v), Glutamax (**#**35050–038, Thermo Fisher Scientific, UK, 1% v/v), and penicillin/streptomycin (**#**15140–122, Thermo Fisher Scientific, UK, 1% v/v).

Female BALB/c mice (8–10 weeks, 20 ± 2 g, n = 25, Charles River Laboratories, UK) were subcutaneously injected with 4T1 cells (2 × 10^6^ cells/100 μL of PBS) in the right flank. 7–10 days after the injection of the 4T1 cells, tumor volume reached approximately 100 mm^3^.

4T1 tumor-bearing mice were randomly allocated into five groups, with five mice in each group. The untreated group served as a general control. In the second group, mice received intratumoral injections of 30 μL Ac-HSA-PLGA-AuNC dispersion (40 mg/mL in saline) without NIR irradiation. Mice in the third group were intratumorally injected with 30 μL of 0.9% NaCl saline and irradiated at the tumor site using an NIR laser (808 nm, 4.5 W/cm^2^) for 10 min. Raw data for the third group are reused here from our previous work.^44^ In the fourth group, mice received 30 μL of Ac-HSA-PLGA-AuNC dispersion (40 mg/mL in saline) intratumorally, followed by NIR laser irradiation (808 nm, 4.5 W/cm^2^) at the tumor site for 10 min. In the final group, mice were treated identically to the fourth group on day 0 and additionally received an intravenous injection of Ac-HSA-PLA (PTX) at a PTX-equivalent dose of 160 mg/kg on day 1.

The detailed method for applying NIR laser irradiation can be found in our previous work.^44^ Tumor diameters (both length and width) were measured daily using a vernier caliper. The formula below was used to calculate the tumor volume. Upon reaching a tumor volume of approximately 600 mm^3^, or in cases where the loss of body weight exceeded 15%, or if tumor ulceration persisted for more than 3 days, the mice were promptly euthanized.

$$\mathrm{Tumor}\;\mathrm{volume}=0.5\times {\mathrm{length}}^{2}\times {\mathrm{width}}^{2}$$

### 4.9 Histology

At the end of the *in vivo* antitumor study, mice in saline group, hyperthermia group and hyperthermia plus chemotherapy group were killed for histopathology imaging. Briefly, mice were anaesthetized and underwent heart perfusion of 20 mL 4% paraformaldehyde neutral solution. Tumors and organs of hearts, livers, spleens, lungs and kidneys were then extracted and respectively stored in 10% neutral formalin solution. These fixed tumors and organs were then embedded with paraffins and sectioned at a thickness of 4 μm. These prepared sections were stained with hematoxylin and eosin (H&E) and imaged.

### 4.10 Statistics

Data are presented as the mean ± SD (standard deviation). The Student’s *t* test was applied in the statistical analysis of biodistribution study and body weight comparison. Two-way ANOVA was used for the statistical analysis of tumor volume comparison. All statistical analyses were performed with Prism software (GraphPad Software, US). A value of *p* < 0.05 indicates a significant difference.

## Supplementary Material



## Data Availability

Data will be made available on request from the corresponding author.

## Notes

UV–vis characterization during Ac-HSA-PLGA-AuNC formulation optimization and additional TEM images of AuNPs excreted in mouse urine (PDF)
