# Supplementary material for: A Renally Excretable Gold Nanoparticle Oncology Platform Enabling Effective Photothermal Therapy and Chemotherapy Combination
Source: ACS Nano Med. 2026 Jan 7;1(2):466–81. doi: 10.1021/acsnanomed.5c00114 (PMC12983353; doi:10.1021/acsnanomed.5c00114)
Supplement: Supplementary file 1 [file nm5c00114_si_001.pdf]

Supporting Information

A Renally Excretable Gold Nanoparticle Oncology Platform Enabling Effective Photothermal Therapy and Chemotherapy Combination

Guojun Xiong[1], Alexandra Vaideanu[1], Ryan D. Mellor[1], Chengwei Jiang[1], Benjamin Gardner[2], Nick Stone[2], Andreas G. Schätzlein[1,3], Ijeoma F. Uchegbu[1,3,4]*

[1] UCL School of Pharmacy, University College London, London, WC1N 1AX, United Kingdom

[2] School of Physics and Astronomy, University of Exeter, Exeter, EX4 4QL, United Kingdom

[3] Nanomerics Ltd., London, EC2Y 5AU, United Kingdom

[4] Wolfson College, University of Cambridge, Cambridge, CB3 9BB, United Kingdom

* Corresponding author: Ijeoma F. Uchegbu; Contact: ijeoma.uchegbu@ucl.ac.uk

Other authors' e-mails:

Guojun Xiong: guojun.xiong@ucl.ac.uk

Alexandra Vaideanu: a.vaideanu@ucl.ac.uk

Ryan D. Mellor: ryan.mellor@ucl.ac.uk

Chengwei Jiang: chengwei.jiang@ucl.ac.uk

Benjamin Gardner: B.Gardner@exeter.ac.uk

Nick Stone: N.Stone@exeter.ac.uk

Andreas G. Schätzlein: a.schatzlein@ucl.ac.uk

Supplementary Figures

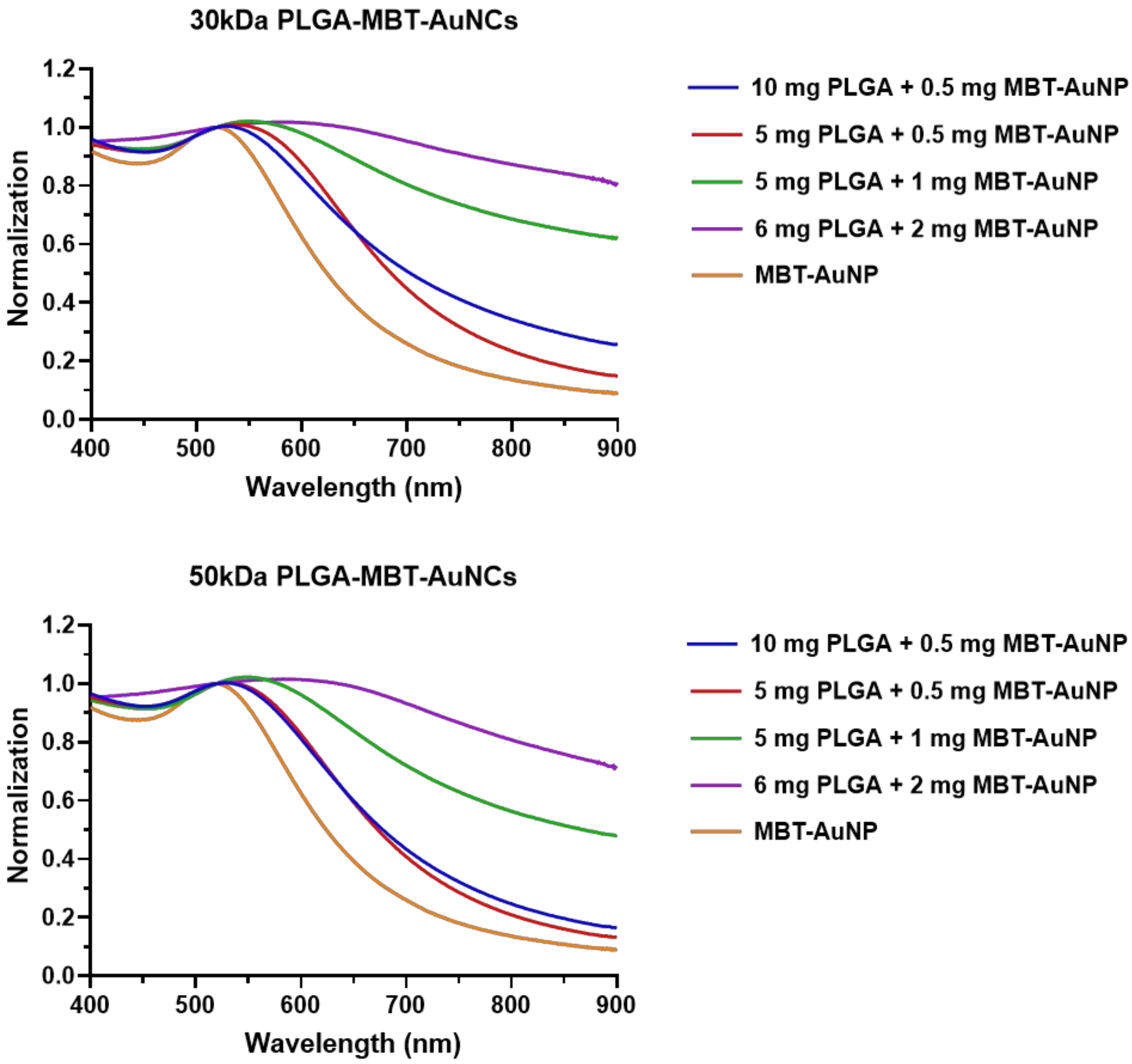

Figure S1. UV-vis absorption of PLGA-mediated clusters of MBT-AuNPs (PLGA-MBT-AuNCs) formed with different PLGA molecular weights.
Both 30 kDa and 50 kDa PLGA were able to cluster MBT-AuNPs, leading to enhanced NIR absorption with increasing MBT-AuNP content.

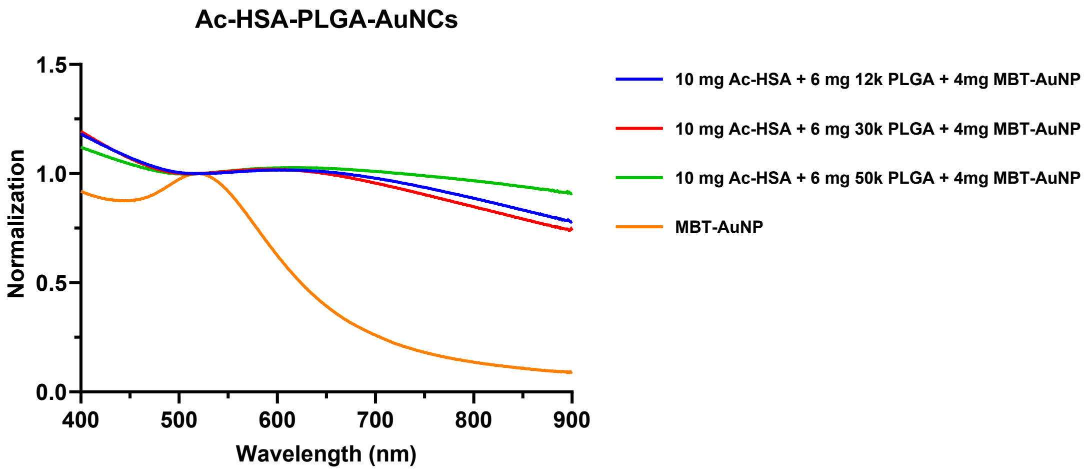

Figure S2. UV-vis absorption spectra of Ac-HSA-PLGA-AuNCs prepared with PLGA of different molecular weights (12 kDa, 30 kDa, and 50 kDa) compared with MBT-AuNPs. All tested PLGA (12, 30, and 50 kDa) could be coated with Ac-HSA to form Ac-HSA-PLGA-AuNCs, and the resulting nanoclusters consistently exhibited enhanced NIR absorption relative to free MBT-AuNPs.

Urine TEM

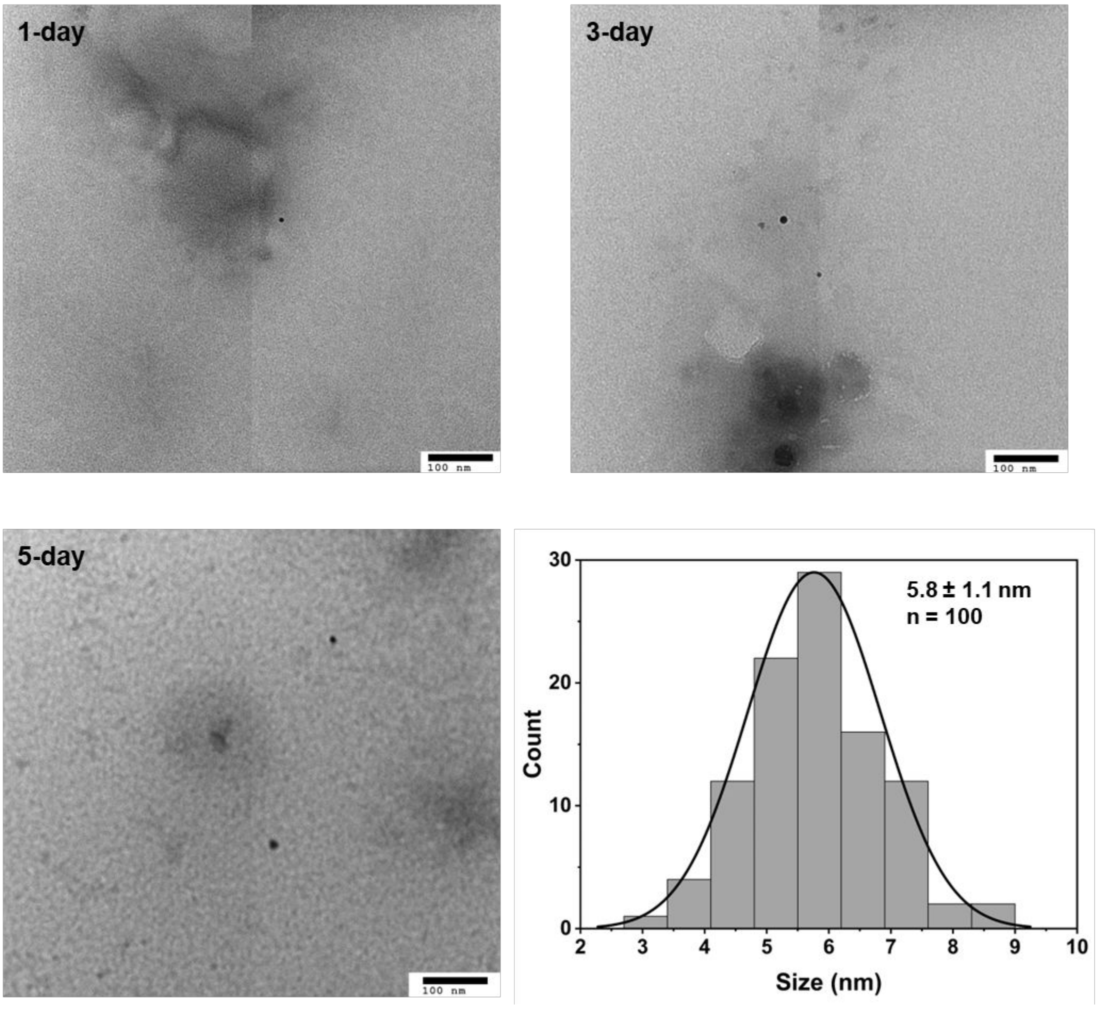

Figure S3. TEM images of ultrasmall AuNPs excreted in mouse urine after intravenous injection of Ac-HSA-PLGA-AuNCs.

Representative TEM micrographs of urinary AuNPs collected at 1-, 3-, and 5-days post-injection are shown. The particles are clearly visible as dark dots with sizes in the ultrasmall range. The histogram (n = 100) indicates a mean particle diameter of 5.8 ± 1.1 nm, confirming that the excreted species are ultrasmall AuNPs.
